# Modeling biominerals formed by apatites and DNA

**DOI:** 10.1186/1559-4106-8-10

**Published:** 2013-04-08

**Authors:** Guillermo Revilla-López, Jordi Casanovas, Oscar Bertran, Pau Turon, Jordi Puiggalí, Carlos Alemán

**Affiliations:** 1 Departament d’Enginyeria Química, E. T. S. d’Enginyeria Industrial de BarcelonaUniversitat Politècnica de CatalunyaDiagonal 64708028BarcelonaSpain; 2 Departament de Química, Escola Politècnica SuperiorUniversitat de Lleidac/ Jaume II nº 69E-25001LleidaSpain; 3 Departament de Física AplicadaEEI, Universitat Politècnica de CatalunyaPça. Rei 1508700IgualadaSpain; 4 B. Braun Surgical S.A.Carretera de Terrasa 12108191Rubí (Barcelona)Spain; 5 Center for Research in Nano-EngineeringUniversitat Politècnica de CatalunyaCampus Sud, Edifici C’, C/Pasqual i Vila s/nE-08028BarcelonaSpain

**Keywords:** Biominerals, DNA, Encapsulation, Hydroxyapatite, Nanopores, Nucleation

## Abstract

Different aspects of biominerals formed by apatite and DNA have been investigated using computer modeling tools. Firstly, the structure and stability of biominerals in which DNA molecules are embedded into hydroxyapatite and fluoroapatite nanopores have been examined by combining different molecular mechanics methods. After this, the early processes in the nucleation of hydroxyapatite at a DNA template have been investigated using molecular dynamics simulations. Results indicate that duplexes of DNA adopting a B double helix can be encapsulated inside nanopores of hydroxyapatite without undergoing significant distortions in the inter-strand hydrogen bonds and the intra-strand stacking. This ability of hydroxyapatite is practically independent of the DNA sequence, which has been attributed to the stabilizing role of the interactions between the calcium atoms of the mineral and the phosphate groups of the biomolecule. In contrast, the fluorine atoms of fluoroapatite induce pronounced structural distortions in the double helix when embedded in a pore of the same dimensions, resulting in the loss of its most relevant characteristics. On the other hand, molecular dynamics simulations have allowed us to observe the formation of calcium phosphate clusters at the surface of the B-DNA template. Electrostatic interactions between the phosphate groups of DNA and Ca^2+^ have been found to essential for the formation of stable ion complexes, which were the starting point of calcium phosphate clusters by incorporating  from the solution.

**Electronic supplementary material:**

The online version of this article (doi:10.1186/1559-4106-8-10) contains supplementary material, which is available to authorized users.

## Background

Hydroxyapatite (HAp), a mineral with formula Ca_10_(PO_4_)_6_(OH)_2_ and hexagonal symmetry, is the most stable form of calcium phosphate at room temperature and in the pH range of 4–12 [1]. This mineral, which is the main component of bones and teeth, is considered as an important biomaterial since several decades ago [2]. Thus, due to both its outstanding biological responses to the physiological environment and its very close similarity to natural bone structure, HAp is currently applied in biomedicine. For example, it is used as a bioactive and osteoconductive bone substitute material in clinical surgery [3, 4], and as a system for the delivery of antitumor agents and antibodies in the treatment of cancer [5–7]. Furthermore, as HAp also has the advantage of absorbability and high binding affinity with a variety of molecules, it has been also used as a purification platform. For example, HAp is applied for the removal of heavy atoms from waste water [8], and for the separation, extraction and purification of proteins [9] and DNA [10].

DNA/HAp biominerals formed by the combination of DNA with a HAp matrix can be viewed from different perspectives. The first refers to the fact that therapeutic DNA is encapsulated in HAp nanoparticles for its subsequent transfection into living cells (*e.g.* liver cells, fibroblasts, osteoblasts and tumor cells) [5–7, 11, 12]. Specifically, HAp nanoparticles with embedded DNA chains can be obtained using different approaches, even though the more popular are those based on the *in situ* precipitation of the inorganic salt in presence of DNA [13, 14] and on the use of a HAp core that is coated using colloidal solutions to give a multi-shell particle [7]. Nanostructured HAp has been shown to be superior for the transfection to other gene delivery methods in terms of immunogenicity and toxicity (*i.e.* safety) [15]. Moreover, the transfection efficiency can be stabilized and enhanced by modulating properties such as extent of DNA binding and encapsulation, particle size, and dissolution behavior of the HAp phases [15, 16]. The interaction between the two entities of the biomineral has been proposed to occur because of the affinity between the calcium of HAp and the phosphate backbone of DNA [17–20]. It should be noted that this proposal makes the nucleotide sequence of DNA unimportant, whereas its length takes major importance.

Another perspective is the one reported by Kostetsky [21], who observed that the period of translation along the *c*-axis of the HAp lattice, 3.4 Å, is relatively similar to the period of the DNA double helix. This feature combined with the fact that the phosphate groups of HAp are able to catalyze the abiogenic synthesis of: D-riboses from ammonia, methane and water [22, 23]; nucleotides from nucleosides condensing agents and ammonium oxalate [24]; and polynucleotides with a 3^′^,5^′^-phosphodiether bond [24] under conditions similar to those of primeval Earth, led Kostetsky to propose a model for the synthesis of DNA through the interaction of its different elements with the lattice of HAp mineral [25]. According to this model, the DNA double helix is embedded into the crystalline network of HAp forming a biomineral similar to that obtained by encapsulating therapeutic DNA into HAp nanoparticles.

On the other hand, in a very recent study Gerdon and co-workers [26] demonstrated the ability of DNA to template the mineralization of calcium phosphate. These authors developed a quartz crystal microbalance sensor for the quantification of HAp formation and the assessment of DNA as a template molecule. The results, which were also supported by optical density and dynamic light scattering measures, FTIR spectroscopy and scanning electron microscopy, suggested that DNA sequesters calcium and phosphate ions, thereby supersaturating the microenvironment and acting as a scaffold on which mineral forms [26]. Moreover, small differences in DNA length, hybridization, and secondary structure were found to provoke differences in affinity for HAp and appear to influence mineralization.

In this work we have used molecular modeling tools to investigate the structure of DNA/HAp biominerals, in which single stranded (ss) and double stranded (ds) DNA are embedded into HAp nanopores. We have focused our analyses on the following aspects: (i) the smallest nanopore size required for the accommodation of ds DNA arranged in the typical B structure [27] (ds B-DNA) during the encapsulation process; (ii) the strain induced by the HAp crystalline field into the B-DNA structure; (iii) the importance of the chemical nature of the inorganic part of the biomineral in the DNA, which has been investigated by comparing DNA/HAp with the biomineral constructed by combining DNA with fluoroapatite [Ca_10_(PO_4_)_6_F_2_, abbreviated FAp], hereafter denoted DNA/FAp; (iv) the encapsulation of ss DNA in terms of molecular strain and relative stability with respect to ds DNA; and (v) the stability of HAp crystals formed around the ds B-DNA core. Furthermore, in order to contribute to a better understanding of the interaction of B-DNA with HAp at the atomic level, we have performed a simulation study of the nucleation and crystal growth of HAp at the ds B-DNA matrix. More specifically, we have used Molecular Dynamic (MD) simulations to provide detailed atomistic models for the initial stages of nucleation and cluster formation of calcium phosphate at a B-DNA molecule. The rest of the paper has been organized as follows. In the next section, we briefly summarize the main characteristics of the crystal structures of natural HAp and FAp, and the chemical structures of the three B-DNA duplexes studied in this work. After this, the strategy and computational details used for both the encapsulation of DNA in apatites and the early processes in the nucleation of HAp at a B-DNA template are described. The results have been organized in several sub-sections, which are devoted to the encapsulation of ds and ss DNA in both HAp and FAp, the validation of the force-field to reproduce organic···inorganic interactions in biominerals, and both the modeling and dynamics of HAp crystals growth around the ds B-DNA template. Finally, conclusions are summarized in the last section of the article.

### Structures of apatites and DNA

Natural HAp has a hexagonal crystal structure with space group P6_3_/m and periods *a*=*b*= 9.42 Å, and *c*= 6.87 Å. The total number of atoms in the unit cell is 44, even though it only contains seven symmetrically independent atoms: two calcium ions, one forming single atomic columns parallel to the *c* axis (Ca_I_) and the other surrounding the hexagonal channel of hydroxyl in groups of the three calcium atoms at different heights (Ca_II_); one phosphor and three oxygen atoms (P, O_I_, O_II_ and O_III_) forming PO_4_ tetrahedral units; and the O(H) ions disordered along *c* about the mirror plane at *z*= ¼. The occupancy of the OH^-^ sites was 50%, as necessary in a P6_3_/m. FAp shows a very similar crystal structure with 42 atoms in the P6_3_/m hexagonal cell (*a*= *b*= 9.40 Å, *c* = 6.88 Å) [28]. In this case, the OH^-^ ions of HAp are replaced by F^-^, which are located on the *c*-axis. The Additional file 1: provides the positions of the non-equivalent atoms used to construct HAp and FAp for the modeling of the biominerals, and the projections of the HAp unit cells.

Three different ds dodecamers, which adopt a B-DNA double helix, have been examined. The chemical structures of the three duplexes, hereafter denoted **I**, **II** and **III**, are 5^′^-CGCGAATTCGCG-3^′^, 5^′^-GCGAGATCTGCG-3^′^ and 5^′^-CGCGAATTC^*^GCG-3^′^, respectively. Sequence **I** is known as the Dickerson’s dodecamer [29] and consists in a well-known sequence with three primary characteristic tracts: CG, AA and TT. Sequence **II** is a conventional sequence that becomes involved in different cellular processes, including carcinogenic ones [30]. Finally, **III** involves methylation of the C5 position of cytidine base, identified as C^*^[31]. This methylation is known to suppress hydrolysis by *Eco*RI restriction enzyme. The 3D structure of the three dodecamers was studied in solution and/or solid state [29–32], a B-DNA arrangement being observed in all cases. Details of the ds B-DNA structure are given in the The Additional file 1: On the other hand, ss DNAs were constructed by removing one of the strands from the three selected duplexes.

### Encapsulation of DNA in apatites and nucleation of HAp

**Figure 1:**
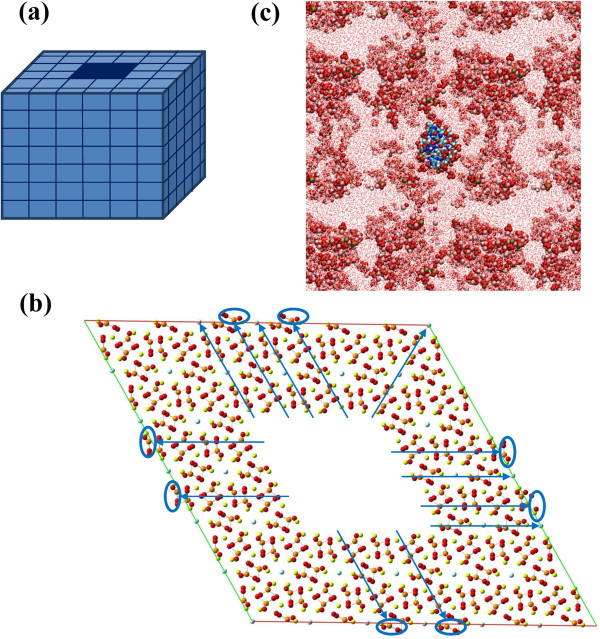
**(a) Simplified scheme of the HAp and FAp super-cells and the hole used to encapsulate ds B-DNA.** (**b**) Atomistic depiction of the FAp super-cell projected onto the (001) plane showing the atoms (blue arrows) and groups of atoms (blue arrows and circles) translated to the border regions of the super-cell. (**c**) Graphical representation of the system used to investigate the initial stages HAp nucleation and growth at a B-DNA in aqueous solution: Dickerson’s dodecamer double helix in the center of the simulation box, which also contains water solvent molecules and Ca^2 +^, 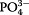 and OH^–^ ions.

Giving the hexagonal symmetry of the two investigated apatites and the molecular dimensions of the B-DNA dodecamers, HAp and FAp models (super-cells) were constructed considering 6×6×7 unit cells. After this, a hole was generated in the center of each super-cell, the dimensions of such hole being defined by the ds B-DNA (*i.e.* steric conflicts between the apatite atoms and the B-DNA were not allowed). After several trials, we found that a hole of 2×2×7 units cells (Figure 1a) was the minimum required to accommodate the double helix without severe steric contacts. In order to completely avoid unfavorable steric interactions between the apatite and the biomolecule, some additional atoms and groups of atoms were translated at their border regions (Figure 1b) allowing us to maintain the electroneutrality of the super-cells. The side length of this hole is 19 Å and its angle γ is 120°. The resulting models (*i.e.* super-cells with a hole of appropriated dimensions at the center) consist of 9856 and 9408 atoms for HAp and FAp, respectively.

The atomic coordinates for the ds DNAs were generated using the NAB (Nucleic Acid Builder) program of the AMBER software [33], which constructed the double helix in the canonical B form. In order to maintain the electrical neutrality of the system, Ca^2+^ ions were put at the minor groove of the double helix, as is frequently observed by X-ray diffraction [34–36]. The positions of these ions were optimized by energy minimization while the coordinates of the rest of the atoms of the system were kept fixed. Models for encapsulated ss DNAs were constructed by removing one of the two strands from the ds B-DNAs embedded in HAp or FAp. In these cases, Ca^2+^ ions were put at random positions around the backbone phosphate groups and subsequently optimized by energy minimization.

The simulation system used to investigate the initial stages of nucleation and cluster formation of calcium phosphate at a B-DNA double helix consisted of the Dickerson’s dodecamer duplex (**I**), which was located at the center of the simulation box, 945 Ca^2+^ ions, 567 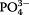 ions, 189 OH^–^ ions, and 29560 water molecules (Figure 1c). In order to provide a comprehensive view of the templating role of the double helix, additional simulations of the same system but without B-DNA were carried out. Several factors are expected to affect the nucleation and growth of the HAp at the B-DNA, ionic concentration being among them. We are aware that the concentration of ions in the simulation systems is higher than in biological conditions. However, modeling much lower concentrations would require computationally prohibitively large simulation boxes. Furthermore, increasing the ion concentration is a practical way to accelerate the simulation of HAp nucleation and growth [37, 38].

## Methods

### Encapsulation of DNA

In order to relax and investigate the stability of the models constructed in the previous section, molecular mechanics calculations based on both energy minimization and MD simulations were applied using the NAMD 2.6 program [39]. Initially, all the models were minimized by applying 5×10^3^ steps of steepest descent to relax the more important conformational and structural tensions. Then, a MD run of 3.0 ns in the NVT ensemble (constant number of particles, volume and temperature) at 298 K was carried out to equilibrate the systems and eliminate small structural tensions. After such thermal relaxation, the saved coordinates were submitted to a new energy minimization by applying 5×10^3^ steps of steepest descent. In both energy minimizations and MD simulation, atoms contained in ds and ss DNAs were the only ones allowed to move from their positions, the coordinates of the mineral being kept fixed at their crystallographic positions in all cases. It should be emphasized that all the systems were calculated in triplicate considering starting points that differ in the orientation of the DNA with respect to the apatite.

The potential energy was computed using the Amber force-field [40, 41]. All force-field parameters for DNA as well as the phosphate and hydroxyl groups of apatites were extracted from Amber ff03 [42]. This is a variant of Amber ff99 [43] in which charges and main chain torsion potentials have been re-derived from quantum mechanical calculations in solution. It should be noted that the ff03 parameters are identical to the ff99-SB [44] ones for nucleic acids, phosphate and hydroxyl groups. Force-field parameters of Ca^2+^ and F^-^ were extracted from the works reported by Bradbrook *et al.*[45] and Dang [46], respectively (see The Additional file 1).

The geometric distortion induced by the apatite in the secondary structure of DNA (Δτ) has been measured as an energy penalty in the bonding contributions using Eqn 1:1
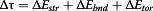


where Δ*E*_*str*_, Δ*E*_*bnd*_ and Δ*E*_*tor*_ refer to the differences in the stretching, bending and torsional energies, respectively. It should be remarked that Δτ exclusively refers to the geometric stress of the double helix, the omission of non-bonding contributions in Eqn 1 allowing us to avoid masking effects associated with strong electrostatic interactions. These differences were calculated by subtracting the energy values associated with the relaxed structure (*i*.*e*. the structure obtained for the DNA embedded in apatite after relaxation by energy minimization and MD simulations) and canonical B-form of DNA (*i*.*e*. the structure directly provided by the NAB module). It should be noted that Δτ accounts for the structural stress induced by the apatite atoms in DNA.

### Validation of the force-field

The reliability of the force-field parameters used in this work to reproduce apatite···DNA interactions in biominerals has been evaluated by comparing the interaction energies derived from molecular mechanics and quantum mechanics calculations. A total of 22 small model complexes containing a fragment of DNA and a fragment of apatite were taken from the modeled biominerals. These complexes, which involved a number of atoms ranging from 53 to 98, were selected to cover the modeling of both attractive and repulsive interactions. The interaction energies, which were estimated as the difference between the total energy of the complex and the energies of the isolated fragments, were calculated using both the AMBER force-field and the B3LYP/6-31G(d) [46–48] quantum mechanical method. The basis set superposition error (BSSE) of the interaction energies calculated at the B3LYP/6-31G(d) level was corrected using the counterpoise (CP) method [49]. All quantum mechanical calculations were performed using the Gaussian 09 computer program [50].

### Nucleation of HAp

MD simulations in NPT conditions (constant number of particles, temperature of 298 K and pressure of 1 atm) were performed using the NAMD 2.6 [39] code to investigate the process of formation and growth of HAp around ds B-DNA molecule in a bath of water molecules. Before the incorporation of DNA, the density of the water in the simulation box was 1.00 g/cm^3^ at a temperature of 298 K. The force-field parameters for DNA, phosphate and hydroxyl groups, and Ca^2+^ were identical to those for the encapsulation study. The water molecules were represented using the TIP3P model [51]. The initial simulation box (92.0×91.5×108.0 Å^3^) was equilibrated using the following strategy. Before any MD trajectory was run, 5×10^3^ steps of energy minimization were performed in order to relax conformational and structural tensions. Next, different consecutive rounds of short MD runs were performed in order to equilibrate the density, temperature, and pressure. First, solvent and ions were thermally relaxed by three consecutives runs, while the B-DNA was kept frozen: 0.5 ns of NVT-MD at 500 K were used to homogeneously distribute the solvent and ions in the box. After this, 0.5 ns of isothermal (298 K) and 0.5 ns isobaric (1 atm and 298 K) relaxation were run. Finally, all the atoms of the system were submitted to 0.15 ns of steady heating until the target temperature was reached (298 K), 0.25 ns of NVT-MD at 298 K (thermal equilibration) followed by 0.5 ns of density relaxation (NPT-MD).

Atom pair distance cut-offs were applied at 16.0 Å to compute the van der Waals interactions. In order to avoid discontinuities in the Lennard-Jones potential, a switch function was applied to allow a continuous decay of the energy when the atom pair distances are larger than 14.0 Å. For electrostatic interactions, we computed the non-truncated electrostatic potential throughout Ewald Summations [52]. The real space term was determined by the van der Waals cut-off (16 Å), while the reciprocal term was estimated by interpolation of the effective charge into a charge mesh with a grid thickness of 5 points per volume unit, *i*.*e*. Particle-Mesh Ewald (PME) method [52]. Both temperature and pressure were controlled by the weak coupling method, the Berendsen thermobarostat [53]. The relaxation times used for the coupling were 1 and 10 ps for temperature and pressure, respectively. Bond lengths were constrained using the SHAKE algorithm [54] with a numerical integration step of 1 fs. Periodic boundary conditions were applied using the nearest image convention, and the nonbonded pair list was updated every 1000 steps (1 ps). The end of the density relaxation simulation was the starting point of the 10 ns production simulations presented in this work. The coordinates of all the production runs were saved every 500 steps (1 ps intervals).

## Results and discussion

### Embedding double stranded B-DNA in hydroxyapatite

**Figure 2:**
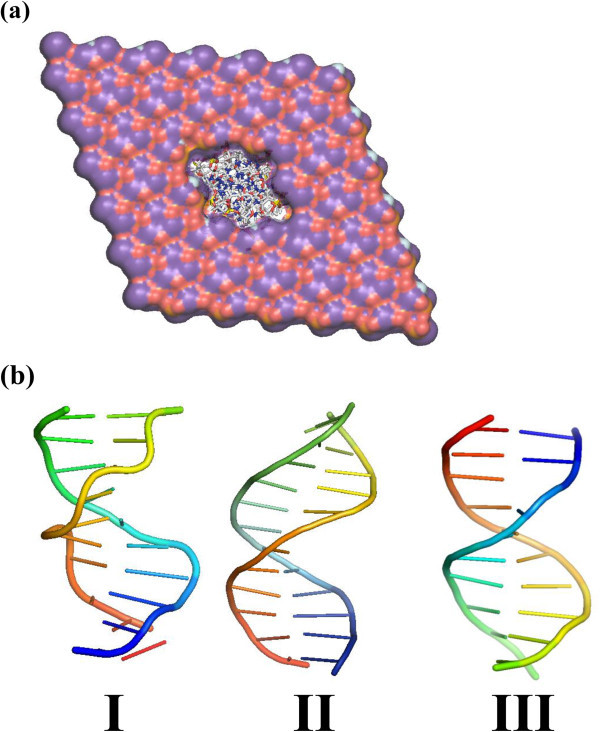
(**a**) **Equatorial perspective of the B-DNA double helix (I) embedded in HAp before relaxation.** (**b**) Axial perspective of the double helix of **I**, **II** and **III** after relaxation.

Figure 2a represents the structure of Dickerson’s dodecamer (sequence **I**) embedded in HAp before relaxation. As it can be seen, the B-DNA occupies practically the whole pore, indicating that its dimensions are appropriated to accommodate the double helix. Figure 2b depicts the double helix after complete relaxation through energy minimizations and MD. Although interactions with HAp atoms induce some distortions in the backbone of B-DNA, both the intra-strand stacking and the inter-strand hydrogen bonds are clearly preserved. The influence of the sequence on the B-DNA distortion induced by the mineral was examined by considering the double helices of **II** and **III** embedded in the same pore of HAp. Inspection of the relaxed structures, which are also included in Figure 2b, indicates that, as observed for **I**, apparently the initial double helices do not undergo significant distortions. This result is fully consistent with previous suggestions, which attributed the binding between HAp and ds DNA to the attractive interaction between the Ca^2+^ ions of the former and the PO_4_^3-^ groups of the latter [17–20]. According to this feature, the role of the nucleotide sequence is relatively unimportant and the stability of the B-DNA inside the pore is essentially due to the dimensions of the latter.

The distortion induced by the mineral in the double helix of each of the three investigated sequences was quantified using the parameters displayed in Table 1, which correspond to: (*i*) Δτ, which measures energy differences of the bonding contributions (Eqn 1); (*ii*) the root mean square deviation (RMSD) between the canonical double helix (*i*.*e*. the starting structure) and the relaxed double helix; and (*iii*) the inter-chain distance (IC) measured with respect to the centers of mass of each strand. Interestingly, the Δτ obtained for **I** was one order of magnitude higher than for **II** and **III**, even though the difference between the bonding contributions was attractive in all cases. Thus, distortion of the backbone produces an energy penalty in the torsional energy but favorable stretching and, especially, bending contributions, resulting in an attractive Δτ value. However, the unfavorable torsional energy contributions, which reflect the conformational distortions induced by the mineral in the canonical B-DNA, are relatively small. The RMSDs ranged from 1.7 to 4.2 Å, which are relatively low considering that they were derived using all the atoms of ds DNA. Finally, the IC showed very small distortions (*i*.*e*. ranging from −0.5 to +2.8 Å) since the IC of the starting structures varied from 19.0 to 20.3 Å, depending on the sequence.

**Table 1:** **Stress induced by the minerals in the DNA molecules (Δτ; in kcal/mol), stretching, bending and torsional contributions to the stress (Δ*E***_***str***_**, Δ*E***_***bnd***_**and Δ*E***_***tor***_***,*respectively), inter-chain distance (IC; in Å) and root mean square deviations (RMSD; in Å) between the initial and the relaxed conformations of the biomolecule for different systems encapsulated in HAp and FAp**

	Δτ ^a^	Δ***E***_***str***_	Δ***E***_***bnd***_	Δ***E***_***tor***_	IC ^b^	RMSD ^c^
	HAp···ds B-DNA					
**I**	−277	−32	−291	+46	22.8	3.5
**II**	−2460	−311	−2160	+11	19.8	1.7
**III**	−1975	−228	−1903	+156	18.7	4.2
	FAp···ds B-DNA					
**I**	+698	+961	−385	+122	17.6	9.7
	HAp···ss DNA					
**I**	−239	−22	−236	+19.2	-	10.3
	FAp···ss DNA					
**I**	−204	−18	−234	+48	-	11.1

^a^ Eqn (1). ^b^ Calculated with respect to the center of masses of each strand. ^c^ The RMSD was calculated considering all the atoms of the duplexes.

The overall of these results indicates that the cavity displayed in Figure 1b allows encapsulate B-DNA double helices without produce mineral-induced stress. Moreover, the dimensions of the pore combined with the crucial role played by the Ca^2+^ are appropriated to avoid any dependence on the nucleotide sequence. Accurate definition of the cavity is provided by the maximum distances between pairs of atoms of the mineral at the internal diagonals of the pore, which are 30.5 and 21.1 Å.

### Embedding double stranded B-DNA in fluoroapatite

**Figure 3:**
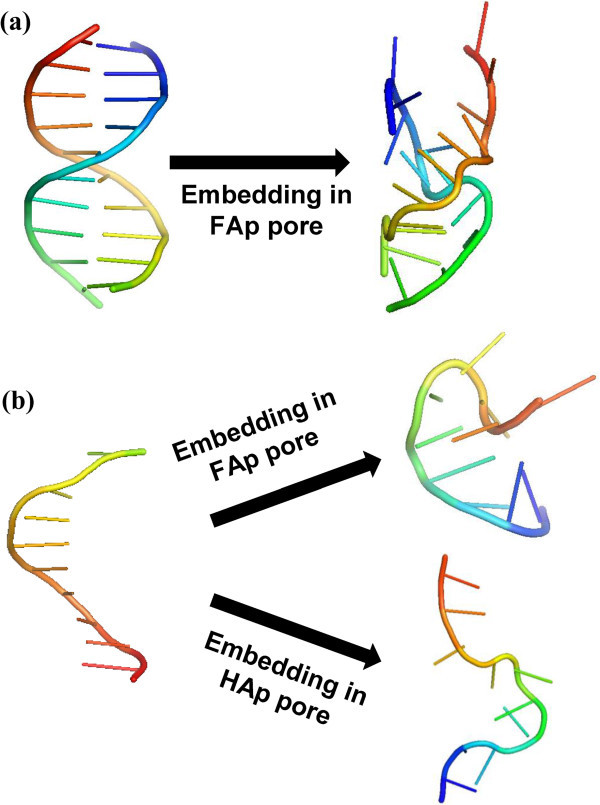
Axial perspectives of (a) the double helix of I before and after relaxation in FAp and of (b) a single strand of I before and after relaxation in FAp (top) and HAp (down).

Relaxation of ds Dickerson’s dodecamer embedded in FAp led to the structure displayed in Figure 3a. Although the general shape of the double helix is retained after energy minimizations and MD, it underwent geometric distortions that affected significantly both the inter-strand hydrogen bonds and the intra-strand π-stacking. Thus, the RMSD calculated with respect to the canonical B-DNA used as starting point was 9.7 Å (Table 1), this value being significantly higher than those obtained for complexes with HAp. Moreover, the energy penalty is repulsive, Δτ= 698 kcal/mol, evidencing the significant geometric stress induced by FAp in the double helix. This stress is essentially concentrated in the bond lengths, the Δ*E*_*str*_ being not only repulsive but also significantly higher than the Δ*E*_*bnd*_ and Δ*E*_*tor*_ contributions. Finally, it should be noted that the repulsive interactions between the FAp and the double helix produces a significant reduction in the IC with respect to the initial value. Thus, the distance between the centers of masses of the two strands in the canonical B-DNA form of **I** was 20.0 Å, decreasing to 17.6 Å after relaxation inside of the FAp pore. This contraction is in opposition with the expansion of 2.8 Å observed when **I** was embedded in HAp, reflecting the repulsive force exerted by fluorine atoms of FAp in the double helix.

### Embedding single stranded DNA in apatites

As expected, ss DNA molecules encapsulated in HAp and FAp underwent drastic conformational changes upon relaxation. Obviously, this should be attributed to the lack of inter-strand hydrogen bonding, which facilitates the distortions induced by the interactions with the mineral. Figure 3b reflects such important variations for ss-**I**. The RMSD obtained for the strand encapsulated in HAp and FAp after relaxation is 10.3 and 11.1 Å, respectively, suggesting that distortions are similar in both cases. The latter is corroborated by Δτ values (Table 1). Thus, the pore is large enough to minimize repulsive interactions, independently of the composition of the mineral, through the complete conformational reorganization of the biomolecule. The relaxed conformation displayed in Figure 3b should be simply considered as one of the many possible disordered conformational states of the ss DNA molecules (*i*.*e*. the pore allows multiple disordered conformational states for the DNA strand, as was observed during the MD simulation). Similar results were obtained for ss-**II** and ss-**III** (not shown).

### Validation of the force-field

We are aware that the molecular mechanics calculations presented in the above sub-sections were carried out using force-field parameters that were not explicitly designed to investigate the inorganic···organic interactions found in biominerals. Specifically, the parameters used to simulate the phosphate and hydroxyl groups of apatites were extracted from Amber ff03 [41], which was explicitly developed to study the dynamics of proteins and nucleic acid in condensed phases, while those used for Ca^2+^ and F^-^ were set to study the crystallographic structure of monosaccharides [44] and the solvation of LiF in polarizable water [45], respectively. Before to investigate the nucleation of HAp at a B-DNA double helix, we performed quantum mechanical calculations on model systems to demonstrate that such force-field parameters satisfactorily reproduce inorganic···organic interactions. A total of 22 model complexes, each containing a fragment of HAp and a fragment of B-DNA, were taken from the model obtained for **I** embedded in the mineral. These model complexes were selected to cover a wide range of interactions, both attractive and repulsive. The interaction between the HAp and B-DNA fragments was calculated for all the complexes using both the force-field potential (ΔE^FF,i^) and the B3LYP/6-31G(d) quantum mechanical method (ΔE^QM,i^), the CP procedure being applied in the latter to correct the BSSE.

**Figure 4:**
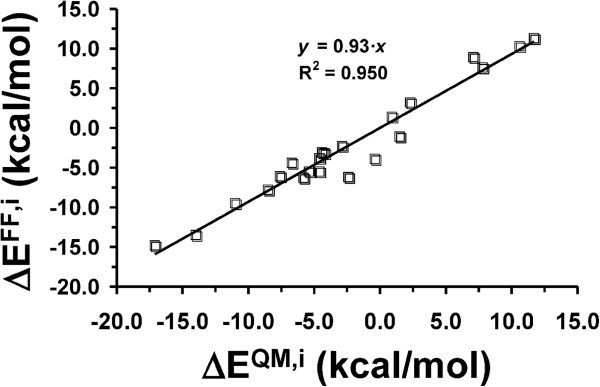
**Representation of the interaction energies calculated for inorganic···organic model complexes using quantum mechanical calculations at the B3LYP/6-31G(d) level (ΔE**^**QM,i**^**) and the AMBER force-field (ΔE**^**FF,i**^**).**

The ΔE^QM,i^ against ΔE^FF,i^ values range from 11.7 to −17.1 kcal/mol and from 11.3 to −14.8 kcal/mol, respectively. Figure 4, which represents ΔE^QM,i^ against ΔE^FF,i^, evidences a close agreement between the force-field and quantum mechanical estimates. Thus, the root mean square deviation between the interaction energies provided by the two methodologies only amounts 1.6 kcal/mol, while the ratio ΔE^FF,i^/ΔE^QM,i^ and the regression coefficient R^2^ are 0.93 and 0.950, respectively. These results demonstrate the ability of the force-field parameters used in this work to reproduce inorganic···organic interactions in biominerals with an accuracy better than expected.

### Nucleation of apatites using B-DNA

**Figure 5:**
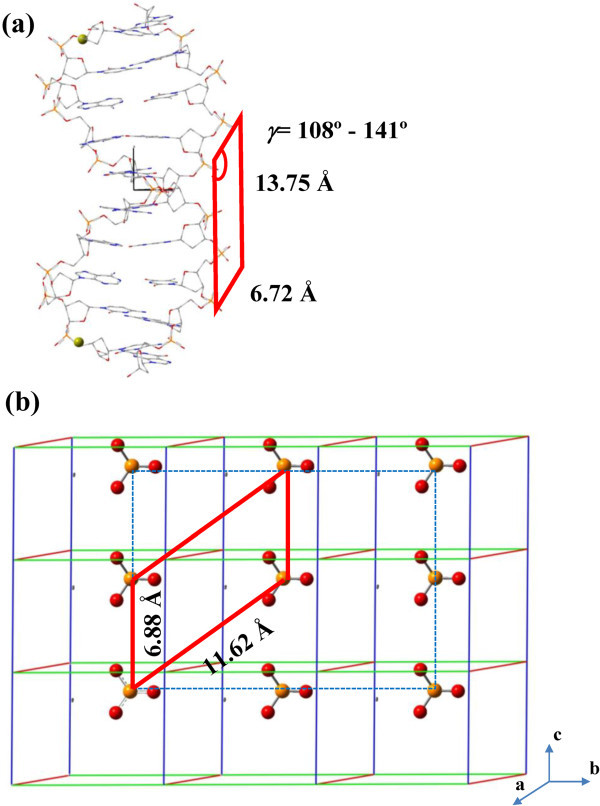
Isomorphic planes identified in (a) the double helix of B-DNA and (b) HAp.

Detailed comparison of B-DNA and apatites structures allowed us to identify a plane defined by four phosphate groups that are located at similar distances in the two systems (Figure 5). Specifically, for HAp / FAp such plane is defined by *u*= 6.87 / 6.88 Å (*i*.*e*. the crystallographic parameter c) and *v*= 11.66 / 11.62 Å, with an angle γ of 120º; while some variability is observed for B-DNA depending on the sequence. Thus, the average values of the sides are *u*= 6.72 Å and *v*= 13.75 Å, the largest variability being shown by the angle with values ranging from 108° to 141°.

**Figure 6:**
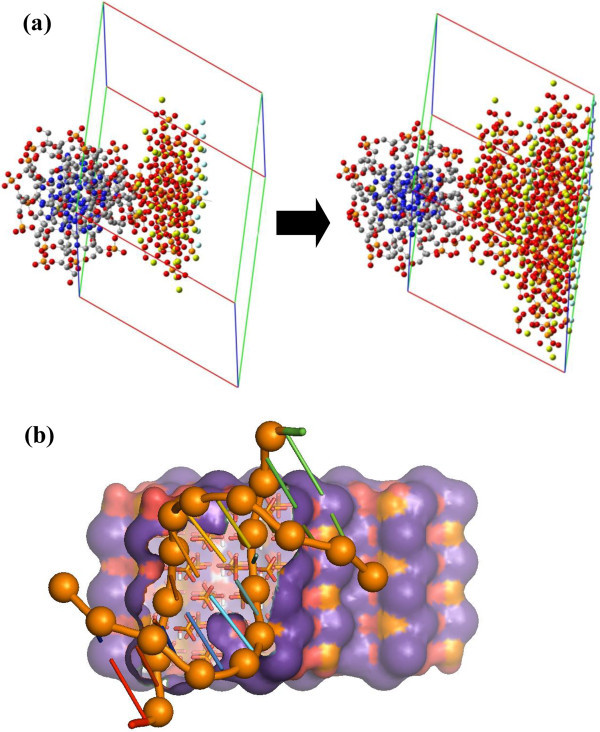
(**a**) **Growing process of HAp using the double helix of B-DNAas a nucleating agent and** (**b**) **biomineral obtained such growing mechanism.**

Starting from the plane identified in sequence **I**, HAp and FAp models were generated from the phosphate groups located at such plane and growing the crystal through the positional parameters displayed in The Additional file 1: Table S1. Accordingly, the four phosphate of the plane identified in B-DNA, as displayed in Figure 5a, were embedded into the apatite crystal. The latter growing process is schematized in Figure 6a for HAp, while Figure 6b displays the resulting structure viewed from a perspective in front of the B-DNA. In order to make understandable the procedure used to build the models, Figure 6 illustrates the procedure considering a single plane of phosphates. The same procedure was applied to build the crystal FAp model (not shown). As suggested from the comparison displayed in Figure 5, we corroborated that the geometric position of the phosphate groups of ds B-DNA is suitable to grow both HAp and FAp crystals. This feature allowed us to conclude that, from a geometric point of view, such biomolecule may act as nucleating agent of the mineral. However, an energy analysis is also required to provide a complete evaluation of this nucleation in the biomineral.

**Figure 7:**
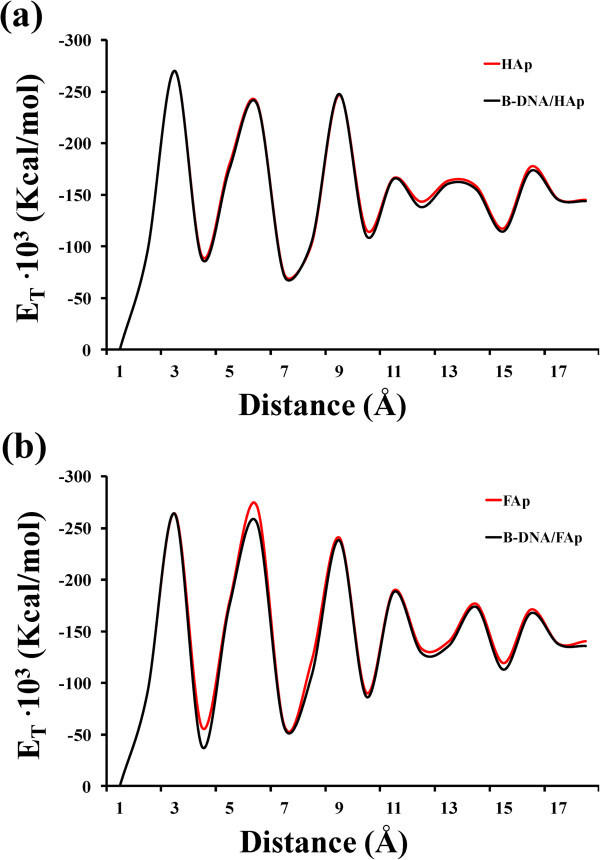
**Variation of the total energy (E**_**T**_**) against the distance from the center of masses of B-DNA for the biomineral and mineral of (a) HAp and (b) FAp.**

Figure 7 compares the variation of the total energy, which is clearly dominated by the electrostatic contribution, of the two biominerals with those of the two minerals as the size of the crystal grows (*i*.*e*. increasing thickness). It should be noted that biominerals refer to the apatite crystals grown around a B-DNA molecule, which is used as a template (*i*.*e*. B-DNA/HAp and B-DNA/FAp) while minerals correspond to pure apatite crystals (*i*.*e*. HAp and FAp without B-DNA). Also, the size or thickness of the crystal is represented by a cutoff distance defined with respect to the center of masses of the phosphate groups belonging to the planes used to nucleate the crystals. The energy profiles obtained for B-DNA/HAp and HAp, which are practically identical (Figure 7a), clearly reflect the lack of influence of the double helix DNA on the energy of the system from the first steps of mineralization (*i*.*e*. mineral thickness < 10 Å). The pattern for B-DNA/FAp and FAp is fairly similar but there is an energetic mismatch between the biomineral and the mineral (Figure 7b), which suggests a stronger distortion of the ds DNA. These results are in agreement with those provided above, which evidenced that FAp distorts more severely the double helix of B-DNA than HAp.

### Dynamics of the initial stages of nucleation of apatites at B-DNA

In order to simulate the early formation and growing of calcium phosphate clusters in the nucleation of HAp at B-DNA double helix template, MD simulations of two systems based on stoichiometric aqueous solutions of Ca^2 +^, 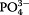 and OH^–^ ions were carried out. In the first system, hereafter denoted HAp/DNA, a double helix B-DNA molecule was immersed in the stoichiometric solution described in the Methods section. The second system, hereafter denoted HAp, no DNA was incorporated to the water box thus remaining the Ca^2 +^, 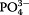 and OH^–^ ions. The analysis of the results was performed on the bases of a 10 ns production run (*i*.*e*. the trajectory after equilibration of the density, temperature, and pressure; see Methods section) in the NPT ensemble. It is worth noting that these simulations are two times larger than those used to examine the early stages of nucleation of HAp at a collagen template [39] and, therefore, the clustering process is expected to occur within such time scale.

**Figure 8:**
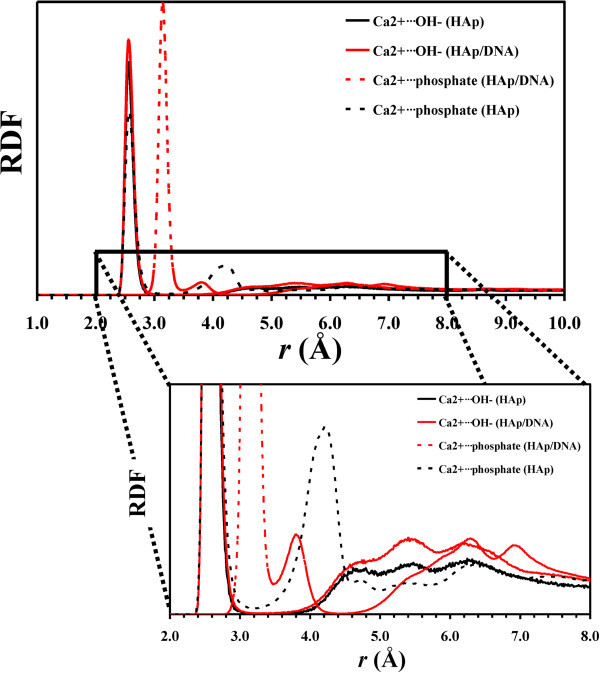
**Radial distribution functions of** Ca^2 +^*···*OH^–^**and**
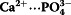
**pairs calculated for HAp and HAp/DNA systems.** Both general and detailed views are displayed.

Figure 8 compares the radial distribution functions (RDF) of Ca^2 +^*···*OH^–^ and 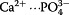 pairs obtained from HAp and HAp/DNA simulations. As it can be seen, the B-DNA template does not have any significant effect in the RDF of the Ca^2 +^*···*OH^–^ pair at distances smaller than 4.5 Å, even though the peaks centered at 5.45 and 6.18 Å are more pronounced in the HAp/DNA system than in the HAp ones. The latter feature indicates that the clustering is higher in the former than in the latter. Differences are more pronounced, especially at short distances, in the RDFs calculated for the 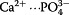 pair in HAp and HAp/DNA. Thus, for the HAp system the first peak appears at 4.22 Å, whereas two well-defined sharp peaks centered at 3.14 and 3.82 Å are clearly identified for the HAp/DNA one. This is consistent with the growing of embryonic clusters in the latter system. Inspection of atomistic configurations extracted from the dynamics indicates that peaks identified for the 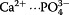 pair at distances lower than 4 Å correspond to 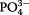 groups surrounded by several (frequently 3) Ca^2+^ atoms.

**Figure 9:**
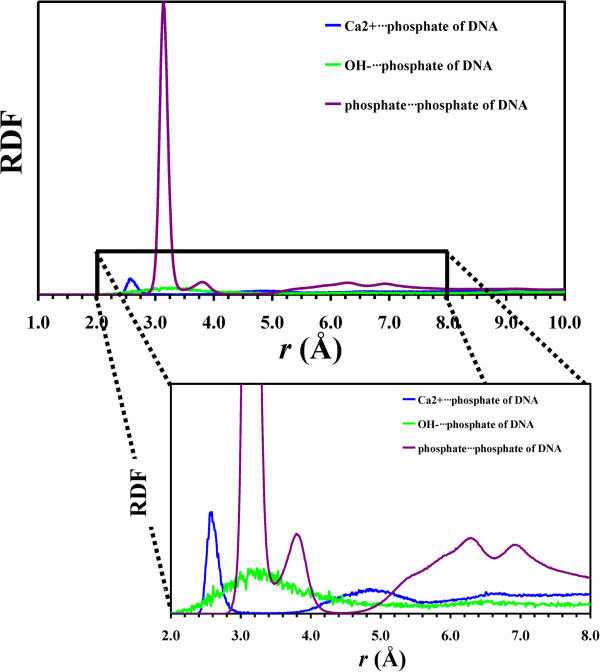
**Radial distribution functions of**
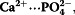

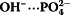
**and**
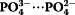
**pairs calculated for HAp/DNA.** Both general and detailed views are displayed.

The nucleation of HAp at the DNA molecule is clearly corroborated in Figure 9, which represents the RDF of 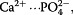

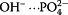
 and 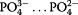 pairs (where 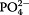 corresponds to the phosphate group of DNA). As it can be seen, the 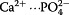 profile shows a peak at 2.58 Å, indicating that the 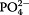 groups of B-DNA are coordinated with Ca^2+^ forming stable ion complexes. Although the role of Ca^2+^ in this complexes may be initially reduced to that of a simple counterion, the peaks observed at 3.14 and 3.84 Å in the 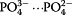 RDF can be only attributed to the nucleation of inorganic crystals at the biomolecule template. Thus, MD simulations reflect that the phosphate groups of DNA interact with Ca^2+^ to form ion complexes, which subsequently coordinate with 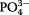 groups of the solution giving place to the stabilization of calcium phosphate clusters at the template. Electrostatic attractions between Ca^2+^ and the phosphate groups of both the DNA and the solution were crucial for the nucleation of the crystals at the template surface. On the other hand, the 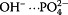 RDF suggests that the role of the OH^–^·is much less decisive in the formation of clusters at the B-DNA surface. Thus, although the profile shows a very broad peak centered at 3.28 Å, it is very poorly defined indicating that the OH^–^ anions are incorporated only occasionally to the calcium phosphate clusters formed at the surface of the B-DNA. Accordingly, the combination of the 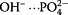 and Ca^2 +^*···*OH^–^ RDFs (Figures 8 and 9, respectively) evidences that the OH^–^remain essentially at the bulk forming complexes and clusters with Ca^2+^. It should be remarked that the low relevance of the OH^–^ anions in the formation of clusters to nucleate HAp crystal at the B-DNA surface is fully consistent with experimental observations. Thus, Tarasevich *et al.*[55] reported that the calcium phosphate clusters act as precursors of HAp (*i.e.* typically octacalcium phosphate) do not incorporate OH^–^ groups.

## Conclusions

In this study, biominerals made of apatite and DNA have been modeled at the atomistic level. The aims of the work were to provide understanding of the influence of the mineral on the conformation of the biomolecule and to determine the possible role of the latter as nucleating agent of the mineral. Encapsulation of the B-DNA double helix in rhombohedric pores (γ= 120°) of HAp does not induce significant structural distortions in the biomolecule. This observation has been found to be independent of the DNA sequence, which has been attributed to the strong stabilizing interactions between the Ca^2+^ atoms of HAp and the phosphate groups of DNA. The minimum dimensions of the pore needed to encapsulate B-DNA are defined by the maximum distances between atoms of the mineral at the internal diagonals, which are 30.5 and 21.1 Å. This result is fully consistent with experimental investigations devoted to encapsulate DNA into HAp nanoparticles, which show a relatively high loading capacity through their pores. The structural stability of the encapsulated double helix is an important finding that offers new possibilities for the design of efficient therapeutic biomedical applications. These results are particularly relevant considering recent findings like the mechanism used by carcinogenic cells to capture HAp nanoparticles [56].

On the other hand, the mechanism proposed for the growing of apatite crystals using the double helix of B-DNA as nucleating agent represents an alternative to classical encapsulation processes (*i.e.* loading of biomolecules at the nanopores and/or the surface). Thus, the controlled formation of biominerals (*i.e.* porous nanoparticles of mineral growing from biomolecules) combined with conventional encapsulation approaches may be used to design therapies with higher efficacy and durability. MD simulations have been used to investigate the initial stages of nucleation and cluster formation of calcium phosphate at a B-DNA double helix in aqueous solution. At room temperature, calcium ions interact with DNA phosphates to form ion complexes, but attracted by electrostatic forces, they coordinate to 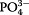 and other calcium ions starting the formation of clusters. Finally, it should be mentioned that this growing mechanism of the biomineral is compatible with the theory and models proposed by Kostetsky [21] to explain the origin of organic matter and life on the Earth.

Additional file 1: Table S1: Positional parameters used to construct the crystal structures of natural HAp and FAp. **Table S2.** Force-field parameters for Ca^2+^ and F^-^. **Figure S1.** Schematic depiction of the unit cell of the investigated apatites (left) projected onto the (001) and (010) planes (top and down, respectively). The hexagonal symmetry is evidenced in the picture at the right, which shows four unit cells projected onto the (001) plane. **Figure S2.** Structure of B-DNA. The double helix is right-handed and makes a turn every 3.4 nm, the distance between two neighboring base pairs being 0.34 nm. Accordingly, there are about 10 nucleotides per turn. The intertwined strands make two grooves of different widths, referred as the *major groove* and *minor groove*. (DOC 735 KB)
