# Supplementary material for: Modeling biominerals formed by apatites and DNA
Source: Biointerphases. 2013 Apr 8;8(1):10. doi: 10.1186/1559-4106-8-10 (PMC6604911; doi:10.1186/1559-4106-8-10)
Supplement: Supplementary file 1 — Additional file 1: Table S1: Positional parameters used to construct the crystal structures of natural HAp and FAp. Table S2. Force-field parameters for Ca2+ and F-. Figure S1. Schematic depiction of the unit cell of the investigated apatites (left) projected onto the (001) and (010) planes (top and down, respectively). The hexagonal symmetry is evidenced in the picture at the right, which shows four unit cells projected onto the (001) plane. Figure S2. Structure of B-DNA. The double helix is right-handed and makes a turn every 3.4 nm, the distance between two neighboring base pairs being 0.34 nm. Accordingly, there are about 10 nucleotides per turn. The intertwined strands make two grooves of different widths, referred as the major groove and minor groove. (DOC 735 KB) [file 13758_2013_8_MOESM1_ESM.doc]

**Supplementary Information**

**Modeling biominerals formed by apatites and DNA**

**Guillermo Revilla-López** (guillermo.revilla@upc.edu)**,^1^ Jordi Casanovas** (jcasanovas@quimica.udl.cat)**,^2^ Oscar Bertran** (oscar.bertran@upc.edu)**,^3^ Pau Turon** (pau.turon@bbraun.com)**,^4,*^ Jordi Puiggali** (jordi.puiggali@upc.edu)**,^1,5^ and Carlos Alemán** (carlos.aleman@upc.edu)**^1,5,*^**

*^1^ Departament d’Enginyeria Química, E. T. S. d’Enginyeria Industrial de Barcelona, Universitat Politècnica de Catalunya, Diagonal 647, 08028 Barcelona, Spain*

*^2^ Departament de Química, Escola Politècnica Superior, Universitat de Lleida, c/ Jaume II nº 69, Lleida E-25001, Spain*

*^3^ Departament de Física Aplicada, EEI, Universitat Politècnica de Catalunya, Pça. Rei 15, 08700 Igualada, Spain*

*^4^ B. Braun Surgical S.A. Carretera de Terrasa 121, 08191 Rubi (Barcelona), Spain*

*^5^ Center for Research in Nano-Engineering, Universitat Politècnica de Catalunya, Campus Sud, Edifici C’, C/Pasqual i Vila s/n, Barcelona E-08028, Spain*

^*^ Corresponding authors: [pau.turon@bbraun.com](mailto:pau.turon@bbraun.com) and [carlos.aleman@upc.edu](mailto:carlos.aleman@upc.edu)

**Table S1.** Positional parameters used to construct the crystal structures of natural HAp and FAp.

| *Atom* | *x* | *y* | ***z*** |
| --- | --- | --- | --- |
| Ca_I_  HAp  FAp | 2/3  2/3 | 1/3  1/3 | 0.0010  0.00144 |
| Ca_II_  HAp  FAp | -0.00657  -0.00712 | 0.24706  0.24227 | 1/4  1/4 |
| P  HAp  FAp | 0.36860  0.36895 | 0.39866  0.39850 | 1/4  1/4 |
| O_I_  HAp  FAp | 0.4850  0.4849 | 0.3289  0.3273 | 1/4  1/4 |
| O_II_  HAp  FAp | 0.4649  0.4667 | 0.5871  0.5875 | 1/4  1/4 |
| O_III_  HAp  FAp | 0.2580  0.2575 | 0.3435  0.3421 | 0.0703  0.0705 |
| O(H) / F  HAp  FAp | 0  0 | 0  0 | 0.1979  1/4 |

**Table S2.** Force-field parameters for Ca^2+^ and F^-^.

| Ion | Mass (g/mol) | σ (Å) | ε (kcal/mol) |
| --- | --- | --- | --- |
| Ca^2+ a^ | 40.90 | 1.7131 | 0.4598 |
| F^- b^ | 19.00 | 3.1680 | 0.2000 |

^a^ From reference 45. ^b^ From reference 46.

**Figure S1.** Schematic depiction of the unit cell of the investigated apatites (left) projected onto the (001) and (010) planes (top and down, respectively). The hexagonal symmetry is evidenced in the picture at the right, which shows four unit cells projected onto the (001) plane.


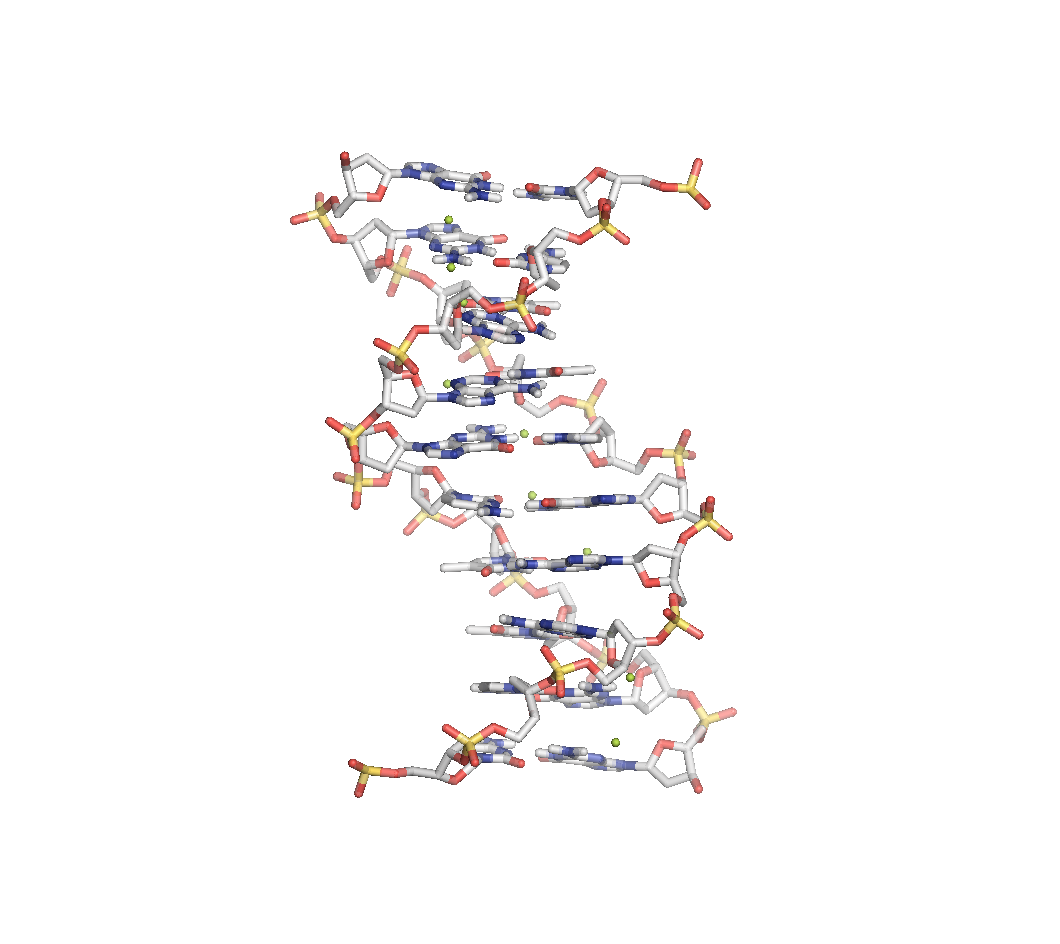


**Figure S2.** Structure of B-DNA. The double helix is right-handed and makes a turn every 3.4 nm, the distance between two neighboring base pairs being 0.34 nm. Accordingly, there are about 10 nucleotides per turn. The intertwined strands make two grooves of different widths, referred as the *major groove* and *minor groove*.
